# Endogenous Retroviruses in Host-Virus Coevolution: From Genomic Domestication to Functional Innovation

**DOI:** 10.3390/genes16080964

**Published:** 2025-08-15

**Authors:** Ruqi Jiang, Jingjun Zhou, Yue Liu, Guanjin Zhou, Dongdong Fan, Lixin Xiang, Ye Chen, Jianzhong Shao

**Affiliations:** 1 Key Laboratory of Cell and Molecular Intelligent Design and Development of Zhejiang Province, College of Life Sciences, Zhejiang University, Hangzhou 310058, China; 3170104883@zju.edu.cn (R.J.); jingjunzhou@zju.edu.cn (J.Z.); zgj18780361913@zju.edu.cn (G.Z.); fandd@zju.edu.cn (D.F.); xianglx@zju.edu.cn (L.X.); shaojz@zju.edu.cn (J.S.); 2 Laboratory for Marine Biology and Biotechnology, Qingdao Marine Science and Technology Center, Qingdao 266071, China

**Keywords:** endogenous retrovirus, evolution, cross-species transmission, KoRV, horizontal transmission, exaptation

## Abstract

Endogenous retroviruses (ERVs) are remnants of retroviral infections that have become stably integrated into host germline genomes. Far beyond passive genomic elements, ERVs actively shape host evolution through complex mechanisms involving genetic innovation, immune modulation, and species adaptation. This review provides a comprehensive synthesis of ERV biology, highlighting recent advances in their classification, amplification mechanisms, and epigenetic silencing. Particular emphasis is placed on the cross-talk between ERVs and exogenous retroviruses (XRVs), demonstrating how receptor competition, recombination, and immune evasion contribute to virus-host co-evolution. We explore ERVs as molecular markers for phylogenetic reconstruction, with case studies such as Koala retrovirus (KoRV) and HERV-K illustrating regional transmission dynamics and co-opted immune functions. Additionally, we discuss the functional domestication of ERVs into regulatory elements, non-coding RNAs, and envelope-derived fusion proteins that influence gene expression, antiviral defense, and placental development.

## 1. Introduction

Endogenous retroviruses (ERVs) are viral elements that remain in the host genome as remnants of retroviruses. These elements originate through the retroviral life cycle, wherein reverse transcriptase (RT) converts viral RNA into DNA, followed by integrase (IN)-mediated genomic integration to form proviruses. Although retroviral infections predominantly affect somatic cells, they can occasionally occur in germ cells or their precursor cells, thereby allowing for vertical transmission to subsequent generations through the germline. Although newly integrated ERVs often retain full coding capacity for viral particle production, evolutionary processes typically lead to progressive functional decay through mutational accumulation. Remarkably, certain ERV components have escaped this degenerative fate, instead being co-opted by host genomes to perform critical cellular functions. These domesticated elements now constitute integral components of host genomic architecture and regulation. The avian leukosis virus (ALV), murine leukemia virus (MLV), and mouse mammary tumor virus (MMTV) were the first ERVs identified through nucleic acid hybridization techniques in two model organisms: chickens (*Gallus gallus*) and mice (*Mus musculus*) [1,2,3,4]. Subsequent advances in molecular genetics and the advent of whole-genome sequencing have revealed the widespread distribution of ERVs across diverse species. It is now well-established that ERVs constitute integral components of all vertebrate genomes, though their genomic abundance varies between species. For instance, ERVs occupy approximately 8% of the human genome [5], ~10% of the mouse genome [3], and exhibit lower proportions in other vertebrates, such as 1.3% in domestic chickens [6] and 2.3% in zebrafish (*Danio rerio*) [7]. These viral remnants serve as molecular fossils that provide critical insights into the origin and evolutionary trajectory of both ERVs and their exogenous retroviral counterparts. This review systematically examines the molecular characteristics and evolutionary dynamics of ERVs, elucidating their contributions to host adaptation via genomic domestication. By integrating cross-species comparative genomics with phylogenetic analyses, we highlight three key biological contributions of ERVs: (1) enhancing host genetic diversity through horizontal transfer events, offering novel perspectives on viral evolution; (2) serving as valuable molecular markers for reconstructing species phylogenies; and (3) functioning as host-domesticated genetic elements that regulate physiological processes. These findings establish a robust theoretical framework for understanding the evolutionary significance of ERVs and their co-evolutionary relationships with host organisms.

## 2. Genome Organization, Classification and Host Distribution of ERVs

The genome organization of ERVs is highly similar to that of exogenous retroviruses (XRVs), typically consisting of two long terminal repeats (LTRs) flanking the *gag*, *pro*, *pol*, and *env* genes. The *gag* gene encodes the structural proteins of the viral particle, including the matrix protein (MA), capsid protein (CA), and nucleocapsid protein (NC), which are crucial for the stability and assembly of the viral particle. The *pol* gene encodes essential enzymes for viral replication, including reverse transcriptase (RT) and integrase (IN), which are responsible for reverse transcribing viral RNA into DNA and integrating the viral DNA into the host genome, respectively. The *env* gene encodes the envelope protein (Env) on the viral membrane, which plays a key role in viral entry into host cells by mediating the fusion of the viral envelope with the host cell membrane. A full-length ERV element typically contains two long terminal repeats (LTRs), located at the 5′ and 3′ ends of the genome. At the time of integration, the 5′ and 3′ LTRs of a full-length provirus are identical due to the mechanism of reverse transcription. Over time, these sequences accumulate mutations independently and diverge from one another. This initial sequence identity allows for homologous recombination between the 5′ and 3′ LTRs, which can result in the deletion ofinternal viral genes and the formation of a solitary LTR (solo LTR). Consequently, in most ERV lineages, the majority of insertions found in host genomes exist in the form of solo LTRs. These solo LTRs represent a more common structural outcome of ERV retention and reflect the genomic decay that occurs over evolutionary time [8]. The length of the LTRs usually ranges from 200 to 1500 base pairs, as more complex LTRs being longer, while degraded or simpler LTRs tend to be shorter. Although the LTR regions themselves do not encode proteins, they contain several regulatory elements essential for viral transcription, such as promoters, enhancers, transcription factor binding sites, splice sites, and polyadenylation signals, which enable the initiation and regulation of viral gene transcription [9]. LTRs are typically composed of three functional regions: the U3, R, and U5 regions. The U3 region contains enhancer and promoter elements, which are primarily responsible for the transcriptional regulation of viral genes. The R region, located between the U3 and U5 regions, serves as the initiation and termination sites for viral transcription. The U5 region includes signal sequences necessary for the integration of the viral genome into the host genome. Through these functional regions, LTRs play a critical role in both the transcription and integration processes of ERVs.

With the increasing number of ERV identifications across various species, the need for systematic classification has become increasingly urgent. Currently, according to the classification standards of the International Committee on Taxonomy of Viruses (ICTV), retroviruses are divided into two subfamilies: Orthoretrovirinae and Spumaretrovirinae [10]. Within this classification, the subfamily Orthoretrovirinae includes six genera: *Alpharetrovirus*, *Betaretrovirus*, *Gammaretrovirus*, *Deltaretrovirus*, *Epsilonretrovirus*, and *Lentivirus*; while the subfamily *Spumaretrovirinae*, commonly known as foamy virus, comprises five genera: *Bovispumavirus*, *Equispumavirus*, *Felispumavirus*, *Prosimiispumavirus*, and *Simiispumavirus* [10]. This classification is based on the characteristics of XRVs. However, compared to XRVs, during long-term evolution after integration into the host genome, ERVs accumulate various sequence variations, such as mutations and deletions [11]. Therefore, traditional retrovirus classification methods alone cannot accurately reflect the characteristics of ERVs. To address this issue, some researchers have focused on the conserved elements encoding RT within the ERV *pol* gene. Based on phylogenetic analysis and referencing similarities with retrovirus classification, ERVs have been divided into three major groups: (1) class I ERVs: primarily comprising γ-retroviruses and ε-retroviruses; (2) class II ERVs: including α-retroviruses, β-retroviruses, δ-retroviruses, and lentiviruses; (3) class III ERVs: including spumaviruses (Table 1) [12,13].

ERVs are widely distributed across various vertebrates [27] and serve as molecular fossils of retrovirus evolution. Among them, γ-retroviruses and β-retroviruses are the most abundant ERV families in vertebrates, prevalent in most vertebrate groups except fish and amphibians, and are particularly common in higher vertebrates such as primates [27]. In contrast, ε-retroviruses are primarily found in fish [28], while α-retroviruses are found in birds and reptiles [28]. δ-retroviruses have only been identified in bats (*Miniopteridae*) so far [21]. Lentiviruses are believed to infect seven mammalian orders: simian, prosimian, feline, bovine, equine, small ruminant, and lagomorph [29]. But to date, endogenous lentiviruses have been identified in only a few species [22,29,30,31]. Spumaviruses, a group of retroviruses with an ancient evolutionary history, trace their origins back approximately 500 million years and exhibit an exceptionally broad distribution. Exogenous spumaviruses are primarily found in various mammalian species [32]. Recent studies have revealed that the endogenization of spumaviruses is remarkably widespread across different species, having been discovered in all vertebrates, including fish, amphibians, reptiles, birds, and mammals [32,33,34,35,36]. Due to their deep evolutionary history and cross-species distribution, these ERVs serve as a crucial research model for exploring the evolutionary processes of viruses.

## 3. Genomic Integration, Amplification, and Epigenetic Silencing of ERVs

The primary source of ERVs is the insertion of XRVs into the host genome. The process of XRV integration into the host genome can be divided into four key steps: viral infection, reverse transcription, nuclear transport of the proviral DNA, and genomic integration. XRVs bind to specific receptors on the host cell surface through the surface subunit (SU) of their Env proteins--for instance, HIV gp120 binding to the CD4 receptor. This receptor engagement triggers a conformational change in the SU-TM complex, leading to the exposure of a hydrophobic fusion peptide within the TM subunit. The fusion peptide subsequently inserts into the target cell membrane. Following insertion, the TM subunit undergoes a characteristic hairpin-like refolding, forming a stable six-helix bundle that draws the viral envelope and host cell membrane into proximity. Ultimately, the juxtaposed lipid bilayers fuse, resulting in the formation of a fusion pore that enables the viral core to be released into the host cell cytoplasm [37]. Next, under the action of reverse transcriptase, viral RNA is reverse transcribed into negative-strand DNA, which is further used as a template to synthesize double-stranded DNA (dsDNA) [38]. Once the double-stranded DNA is formed, integrase processes it into a linear structure suitable for integration and associates it with host proteins to form the pre-integration complex (PIC) [39,40]. The PIC is actively transported through the nuclear pore or enters the nucleus during cell division when the nuclear membrane disassembles [40]. Although integration can occur broadly throughout the genome, different retroviral genera exhibit distinct site preferences. For example, lentiviruses such as HIV-1 preferentially integrate into actively transcribed genes, whereas γ-retroviruses like MoMLV tend to target strong enhancers, active gene promoters, and regions near CpG islands. Other retroviral genera also display unique patterns of integration. Studies have shown that these preferences are largely guided by interactions between viral integrases and specific host cofactors, such as LEDGF/p75 for HIV-1 and BET family proteins for MoMLV [41,42]. Subsequently, the integrase creates staggered cuts in the host DNA and covalently links the proviral DNA to the host DNA [39]. Finally, integration is completed through the host’s DNA repair mechanisms [43]. Notably, due to the lack of proofreading activity in reverse transcriptase, XRVs are prone to high-frequency mutations during reverse transcription, which serves as a significant source of genetic diversity for ERVs [44].

**Figure 1:**
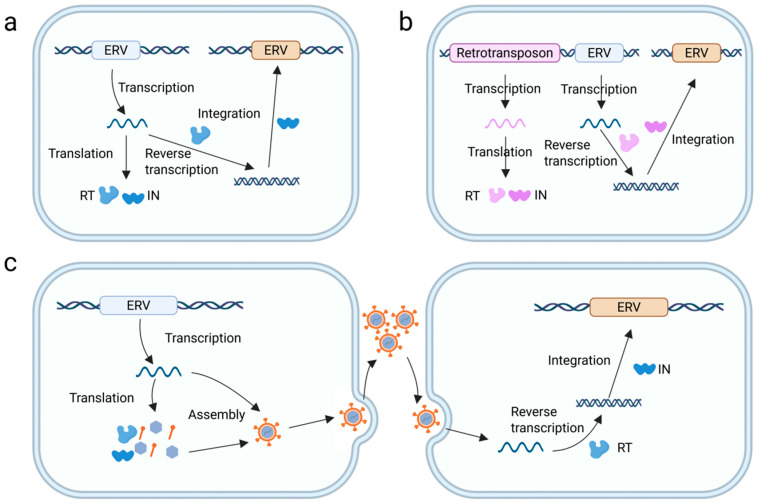
Endogenous replication and amplification of ERVs. (**a**) Autonomous retrotransposition: The retrotransposition of ERVs is mediated by the RT and IN enzymes carried by the virus itself, (**b**) Trans-mediated retrotransposition: The retrotransposition of ERVs is mediated by RT and IN enzymes carried by other endogenous retroviral elements present in the host genome, (**c**) Re-infection of ERVs: ERVs re-infect the host by forming viral particles and inserting ERV sequences at new loci.

In recent years, increasing attention has been given to the amplification mechanisms of ERVs, revealing that their proliferation is not limited to the classical pathway of exogenous viral reinfection but also involves several endogenous mechanisms, though it occurs at a relatively lower frequency (Figure 1). Among these, reinfection is a key route by which ERVs achieve amplification. Under specific conditions, ERVs can produce infectious viral particles that can reinfect host cells. Such reinfection can occur between germline cells or from somatic cells to germline cells, thereby facilitating new germline integration and subsequent expansion [45,46]. In a study of HERV-K, Robert Belshaw et al. found that the copy number of the HERV-K (HML-2) family increased primarily through sustained reinfection events, suggesting that certain ERV families may maintain a long-term, smoldering replication potential throughout evolution [46]. In addition, ERVs can amplify through retrotransposition, which comprises three distinct modes. The first is *cis*-mediated retrotransposition, in which an ERV with intact coding regions for reverse transcriptase and integrase can autonomously synthesize the required enzymatic proteins and integrate new copies into the genome. This mode typically lacks a functional *env* gene; thus, it does not produce infectious particles [47,48]. The second is *trans*-mediated retrotransposition, wherein defective ERVs rely on the enzymatic machinery provided by co-expressed endogenous or exogenous elements, such as other retroviruses or retroelements; notably, this process may sometimes result in the formation of viral particles carrying heterologous genomes and may involve infection [47]. Finally, a third amplification pathway involves retrotransposition via LINE-encoded enzymatic functions. This mechanism is independent of retroviral proteins and enables ERVs to transpose by hijacking the reverse transcriptase of LINE elements [47]. For example, HERV-W elements have been shown to utilize proteins encoded by LINEs to facilitate their amplification, and more than 60% of HERV-W loci appear to have been generated with the assistance of LINE machinery [49].

It is noteworthy that the early originated ERVs exhibited active transcriptional activity in the genomes of early vertebrates, but they transitioned to a highly silenced state in mammals. This indicates the gradual establishment of epigenetic silencing mechanisms during ERV evolution. For example, a total of 3315 ERVs were recently identified in zebrafish, among which approximately 317 exhibited significant transcriptional activity [7]. These active ERVs showed high levels of transcription across different stages of embryonic development and in various adult tissues such as the heart, spleen, intestine, and brain, demonstrating clear developmental stage- and tissue-specific expression patterns. This finding suggests that although the genomic proportion of ERVs in early vertebrates is much lower than that in higher vertebrates, a considerable portion remains transcriptionally active and is involved in regulating multiple biological processes, potentially playing important roles in physiological and developmental contexts. With the progression of species evolution, most ERVs in higher vertebrates have been gradually silenced through epigenetic mechanisms, thereby reducing their potential threat to the stability of the host genome. In mammals, the DNA methyltransferase family DNMT3 is closely associated with the Piwi/piRNA-mediated DNA methylation pathway [50,51]. DNMT3A and DNMT3B can recognize specific sequences in ERV promoters, such as CpG islands in LTRs, and mediate de novo methylation to repress their transcriptional activity [51]. At the level of histone modification, KAP1/TRIM28 and the histone methyltransferase SETDB1 form a multiprotein complex that is recruited to ERV loci, where it deposits H3K9me3 marks, inducing chromatin condensation and ERV silencing [52]. Moreover, recent studies have highlighted the critical role of RNA modifications in ERV silencing, including N6-methyladenosine (m6A) modification of mRNA and 5-hydroxymethylcytosine (5hmC) [53,54,55], both of which can regulate ERV expression at the post-transcriptional level. These findings further expand the complexity of ERV epigenetic regulatory mechanisms and underscore the multi-layered epigenetic control strategies governing ERVs across different evolutionary stages.

## 4. Interactions Between ERVs and XRVs

The interaction between ERVs and XRVs reflects the complex evolutionary arms race between viruses and hosts. Extensive studies have demonstrated significant interactions between ERVs and XRVs at multiple levels. As a classic example, the molecular interaction mechanisms between endogenous sheep retrovirus (enJSRV) and Jaagsiekte sheep retrovirus (JSRV) have been extensively investigated. JSRV is the causative agent of ovine pulmonary adenocarcinoma (OPA) and is horizontally transmitted within populations through the respiratory route [56]. Sheep hyaluronidase 2 (Hyal-2) acts as a common cell surface receptor for both endogenous and exogenous JSRV [57], suggesting that enJSRV may inhibit the invasion of exogenous JSRV through receptor competition, thereby forming a natural antiviral barrier in the host. enJS56A1 and enJSRV-20 (also known as enJSRV-6q13) are two key ERV elements located in the 6q13 region of the sheep chromosome [58]. Phylogenetic analysis indicates that their genomic integration events occurred during the divergence of the *Ovis aries* (sheep) and *Capra* (goat) lineages, approximately 5–11 million years ago [59]. It is noteworthy that the *gag* gene coding regions of these two endogenous viral copies display substantial structural alterations; specifically, a substitution of the highly conserved arginine (R) at position 21 in exogenous JSRV with tryptophan (W) [60]. This domain-specific substitution has been confirmed as a protective mutation with viral suppressive function, capable of forming a Gag protein with dominant negative effects. This protein forms a polymer with JSRV Gag, which is subsequently degraded by the cell’s proteasomal mechanism [61]. Additionally, molecular evolutionary analyses demonstrated that the 6q13 genomic region underwent marked structural expansion during sheep domestication, showing a positive association between the enJSRV-6q13 copy number and the prevalence of protective mutations [58]. This phenomenon suggests that a dosage effect, potentially through the increased expression of the mutant Gag protein, may enhance the host’s resistance to JSRV infection. In addition, the embryo-specific expression of enJSRV may regulate the host immune response through immune tolerance mechanisms, indirectly enhancing the immune surveillance function against JSRV [62]. In contrast, JSRV can replicate by selecting tissue microenvironments with low or no expression of enJSRV, thereby escaping the blocking effect mediated by endogenous viruses [59]. This dynamic balance mechanism profoundly reflects the interaction between selective pressure and adaptive strategies in the host-virus coevolution process. Similar interactions also exist between feline leukemia virus (FeLV) and endogenous feline leukemia virus (enFeLV). FeLV-A can undergo homologous recombination with enFeLV, leading to the emergence of the more pathogenic FeLV-B subtype [63,64,65]. This recombinant subtype is generated through recombination between the FeLV-A and enFeLV *env* regions as well as the 3′ LTR region. FeLV-B infection leads to feline leukemia and the development of lymphoma. On the other hand, studies have also shown that when specific pathogen-free (SPF) cats are infected with FeLV, those with high enFeLV levels are less likely to develop persistent antigenemia [66], suggesting that enFeLV may mitigate FeLV-induced disease.

The interaction mechanism between human endogenous retrovirus K (HERV-K) and human immunodeficiency virus (HIV) is highly complex and has long been a key focus of research on the co-evolution of retroviruses and their hosts. Existing studies reveal that HIV infection can significantly improve the transcriptional activity of HERV-K, but its molecular regulatory network has not yet been fully elucidated [67]. Current investigations indicate the following: (1) HIV-induced CD4^+^ T cell depletion and systemic immune microenvironment disruption may lead to epigenetic repression loss at HERV-K loci, such as through DNA methylation decline and reduced levels of the repressive histone mark H3K27me3 [68], thereby activating HERV-K transcription. (2) The HIV-1 Tat protein activates NF-κB and NF-AT, and these transcription factors directly interact with the HERV-K (HML-2) LTR promoter, further driving HERV-K expression [69,70]. (3) HIV infection can elevate levels of IFN-γ and TNF-α [71], which drive HERV-K LTR-dependent transcription by activating the NF-κB or STAT signaling pathways [72,73]. Notably, HERV-K and HIV exhibit unique interactions at the viral assembly level. Experimental evidence shows that the Gag protein encoded by HERV-K can undergo heterotypic co-assembly with HIV viral components through a domain-complementary mechanism, forming chimeric virion-like particles [74]. Such immune cross-reactivity against HERV-K antigens might stimulate ADCC and HIV-directed CD8^+^ T cell activity, promoting infected cell clearance--highlighting a possible functional interplay between HERV-K and antiviral immunity [75]. However, the double-edged sword effect of this interaction system remains to be evaluated. On one hand, HERV-K antigens may divert the host immune focus away from HIV-specific epitopes through molecular mimicry, facilitating an immune evasion mechanism that allows HIV to escape host immune surveillance and establish persistent infection. On the other hand, the cross-immune response induced by HERV-K may offer new insights for developing broad-spectrum antiretroviral strategies.

## 5. ERVs as Genomic Markers for Tracking Retroviral and Host Evolution

In the study of ERVs from an evolutionary perspective, the estimation of insertion time is a crucial step in analyzing the viral evolutionary trajectory. Several methodologies have been established to determine the integration age of ERVs in the genome, with the LTR-based molecular clock analysis model being the most widely used method. This method primarily relies on the mutation rate of the LTRs within ERVs and the known mutation rate of the host genome to estimate the insertion time of ERVs. Specifically, when an ERV provirus integrates into the host genome, the 5′ and 3′ LTR sequences are identical [76,77]. During host genome evolution, these homologous sequences accumulate genetic variations independently [78]. By calculating the genetic distance (D) between them and incorporating the host-specific mutation rate (μ), the integration time can be estimated using the formula T = D/2 μ [79]. For the human genome, the baseline mutation rate is typically set at 1.61 ± 0.13 × 10^−8^ [80], and this parameter must be adjusted for species-specific differences. However, the LTR molecular clock model has significant limitations in practical applications. The primary issue is that during long-term evolution, structural variations in the LTR regions of some ERV elements can lead to the loss of one LTR or the formation of solo-LTRs [81], rendering this method inapplicable. Additionally, the mutation rates in different functional regions of the host genome exhibit spatial heterogeneity, and ERV integration sites may be influenced by positive or purifying selection, causing local mutation rates to deviate from the genome-wide average [78,82]. Researchers have proposed the following recommendations for applying molecular clock techniques to estimate ERV insertion times: First, using high-precision sequencing technologies to ensure high-quality and high-coverage genomic data, thereby improving genome assembly completeness. Second, conducting systematic sequence alignments of ERV family members, focusing on verifying the sequence integrity of conserved domains and integration sites, and prioritizing ERV elements with intact LTRs for molecular clock modeling [82]. Furthermore, some researchers have improved the traditional LTR molecular clock model by integrating phylogenetic analysis methods. Specific implementation strategies include constructing a maximum likelihood phylogenetic tree based on the known insertion times of LTR sequences and inferring the insertion times of unknown sequences using the known LTR insertion times [78,83,84]. For genome-wide studies, simplified phylogenetic analysis methods can complement the LTR molecular clock model effectively.

By insertion time calculation, the origin of ERVs can be traced back to viral infection events that occurred tens of millions or even hundreds of millions of years ago. These viruses infected host germline cells and integrated their genomes into the host DNA, giving rise to ERVs. Throughout the host’s evolutionary history, ERVs have been stably preserved as “genomic fossils” across different species. Approximately 500 million years ago, the first retroviruses began infecting early vertebrates and integrated into their germline cells, forming the earliest endogenous retroviruses [28]. The emergence of ERVs is characterized by multiple independent integration events, resulting in complex spatiotemporal diversity. For instance, murine ERVs (MuERVs) in rodent genomes have undergone at least three major waves of infection: around 10 million years ago, 30 million years ago, and even earlier periods [85]. Similarly, HERV-K (HML-2) in the human genome experienced major expansion phases driven by recurrent reinfection events over a span of 0.15 to 35 million years [86,87,88]. Interestingly, the colonization of the human genome by β-retroviruses did not occur at a constant rate. Instead, there were distinct bursts of integration activity, likely corresponding to periods of high viral replication, interspersed with prolonged phases of low integration frequency [89]. Although ERV insertion sites are randomly distributed, homologous ERV sequences can still be detected at the same genomic loci in closely related species, indicating that the ERV integration occurred in a common ancestor prior to species divergence and was subsequently retained in descendant lineages through vertical transmission. These evolutionarily conserved ERV insertions serve as molecular markers, offering critical evidence for elucidating phylogenetic relationships among species. Therefore, comparative analyses of ERV insertion site conservation and sequence homology provide a powerful framework for inferring speciation nodes. A classic example is found in primate evolution studies. Chen et al. annotated HML-9 elements in the genomes of gorillas and humans, revealing that these elements integrated into the chimpanzee genome between 14 and 36 million years ago (mya), with an average insertion age of 25 mya, and into the human genome between 18 and 49 mya, averaging 29 mya. Notably, fossil-based estimates place the divergence of humans and chimpanzees at approximately 6.5–7.5 mya, which is significantly later than the estimated integration time of HML-9. This strongly supports the hypothesis that these retroviral elements became endogenized in the genome of a common ancestor prior to speciation [90]. Similarly, the ERV-E family appears to be unique to Equidae (horse-related species), with no homologous sequences detected in primates or other mammalian lineages. Phylogenetic analyses based on long-sequence alignments have elucidated the evolutionary trajectory of this ERV clade within the genus Equus. In particular, the orthologous insertion site of EqERV.b1 in both horse and donkey genomes indicates that the integration event predates their divergence, estimated at 6–10 mya [91]. Using ERVs to infer species divergence nodes offers a novel perspective for studying species divergence. ERV integration is a random event, and the probability of different viruses independently inserting into the same genomic site is extremely low. Therefore, shared ERV insertion sites can serve as strong evidence of common ancestry among species. ERVs can span different species and provide a unique evolutionary timescale, especially for those lacking sufficient fossil records. However, in practical research, genomic recombination, deletion, or mutation may lead to partial or complete loss of ERV sequences. This is particularly true for ancient divergence events, where ERVs may have degraded to the point of being unrecognizable. Additionally, the reliance on complete genome data limits their application in studies of extinct species or ancient DNA. Therefore, combining ERVs with other molecular markers, fossil evidence, or genomic data can yield more accurate estimates of species divergence times.

## 6. ERV Cross-Regional Spread and Host Population Dynamics

When host populations expand, diversify, or migrate, the regional distribution of ERVs can reflect the evolutionary history of the host species. By studying the distribution of ERVs in different regions or host populations, researchers can reconstruct the population structure and evolutionary pathways of the host. The transmission process of retroviruses in different regions involves the combined effects of multiple biological and ecological factors. Firstly, direct or indirect contact between the host and the virus is a fundamental prerequisite for cross-species transmission [92]. In addition, the ability of a virus to evade immune recognition and clearance and successfully integrate into the host genome is a critical step for establishing persistent infection and enabling viral spread [93]. After integration, the virus must also be capable of assembling new viral particles and achieving reinfection, ensuring the cyclical transmission of the virus within the host individual and population. At the ecological level, factors such as the host’s population size, distribution patterns, and interactions with environmental factors influence the virus’s speed and extent of spread [94]. Studies on mice have shown that the prevalence of ERVs can vary from 0% to 42.86% in different wild subpopulations [95], indicating significant differences in the transmission efficiency and patterns of ERVs across different populations or geographic contexts. This underscores the critical role of geographic distribution in the spread of ERVs.

In the field of the regional distribution of ERVs, Koala retrovirus (KoRV) is undoubtedly one of the most representative models. KoRV is a γ-retrovirus associated with chlamydial diseases and has been found to be linked to tumorigenesis in koalas [96,97]. The uniqueness of KoRV lies in its dual biological properties as both an endogenous and exogenous virus. Specifically, the KoRV-A subtype is the only endogenized virus, retaining characteristics of both exogenous and endogenous viruses. It can exhibit stable inheritance patterns within the host chromosomes [98,99,100], while also maintaining the capacity for horizontal transmission [100]. The other 10 subtypes (KoRV-B to K) primarily differ in high-frequency mutation sites within the receptor-binding domain (RBD) of the Env protein [101,102], and these subtypes retain the replication capacity and horizontal transmission potential of exogenous viruses [103]. The transmission of KoRV in koala populations shows significant differences in relation to regional distribution. In the koala populations of Queensland and New South Wales, the KoRV provirus positivity rate is close to 100%, indicating nearly universal infection. In contrast, in the southern regions, such as Victoria and South Australia, the positivity rate significantly decreases, generally falling below 50% [101,104,105,106]. Further molecular testing reveals high copy numbers of proviral DNA in blood and tissue samples from koalas in the northern regions, suggesting active viral replication or recent integration events. In contrast, in the southern regions, some koalas only show low copy numbers of proviral DNA, indicating limited viral replication ability or possible genomic deletions [98,102,107,108]. To explain the mechanisms behind the differential transmission of KoRV in northern and southern koala populations, researchers have proposed several hypotheses. One hypothesis suggests that KoRV initially entered the koala populations in northern Australia and gradually spread southward through horizontal transmission [105]. Koalas have relatively limited migration behavior in their natural habitat, with a small home range primarily concentrated in specific areas. Gene flow between koalas usually occurs through the local migration of individuals. However, the regional distribution differences in KoRV provide direct evidence that koala populations may have experienced a north-to-south expansion, reflecting the historical migration patterns of koalas [105]. Another hypothesis focuses on the large-scale hunting of koalas by European colonists in the late 19th to early 20th century. Koalas in the southern regions were once nearly extinct due to overhunting, and the koala populations in Victoria were used for population restoration in the south [109]. This restoration process was accompanied by a significant loss of genetic diversity, resulting in a population bottleneck effect, which may have led to a reduction in KoRV copy numbers [109]. Recent research has further expanded our understanding of this phenomenon. Studies have revealed that some southern koalas previously considered KoRV *pol*-negative harbor a defective, potentially endogenous KoRV variant termed “RecKoRV” [96,106]. This variant may have arisen from recombination events between KoRV and the host genome or other endogenous retroviruses, resulting in significant sequence deletions or structural changes in its genome [110]. Due to the incomplete genetic structure of these defective viruses, conventional qPCR or RNA sequencing techniques are unable to effectively detect them, creating an apparent “virus-negative” state [106]. This finding suggests that KoRV may have been widely transmitted in koala populations at some point in the past, but due to subsequent recombination events or genomic deletions, it now exists in the southern populations in a residual or defective form. This latent viral legacy could provide a new perspective for understanding the regional distribution differences in KoRV, its endogenization process, and its long-term co-evolution with the host genome.

## 7. Pathways and Barriers in ERV Cross-Species Transmission

Viral cross-species transmission refers to the process by which a virus overcomes species barriers, transferring from its original host to a new host and establishing an effective infection. This process typically involves key steps such as interactions between the virus and the new host’s receptors, immune evasion, and adaptive evolution [111]. ERV cross-species transmission can occur through various mechanisms, with the most common being direct infection by retroviruses. In natural environments, direct contact between organisms, such as through predation or parasitism, as well as indirect contact via shared habitats, respiration, or excretion, can facilitate this process [28,112,113,114]. During these interactions, retroviruses may come into direct or indirect contact with another species suitable for infection, and their viral genomes may integrate into the genome of the new host, thereby becoming a new ERV [114]. Cross-species transmission is relatively common among γ-retroviruses and their associated class I ERVs [115]. Although ERV transmission between taxonomic units at the order level or below (e.g., primates–rodents or primates–chiropterans) is relatively frequent [116], cross-class transmission (e.g., mammals–birds) is extremely rare [115]. This may be related to the conservation of viral receptors, differences in host immune systems, and the efficiency of germline infection [117]. Table 2 lists several well-documented cases of cross-species transmission.

Intermediate hosts are typically located between different species or ecological niches and can facilitate the spread of ERVs from one species to another. The transmission and adaptation of viruses between hosts provide significant evolutionary driving forces. The transmission patterns of the KoRV and gibbon leukemia virus (GaLV) support the intermediate host hypothesis. Although koalas and gibbons are distributed in Australia and Southeast Asia, respectively, and are separated by natural barriers such as the Wallace Line, the nucleotide sequences of these two viruses show a high degree of similarity, and a phylogenetic analysis groups them in the same clade [122]. This suggests that the viruses likely underwent transmission across both species and geographic boundaries. In this context, rodents and bats are considered key intermediate hosts in the cross-species transmission of GaLV and KoRV. A study identified a novel retrovirus (MbRV) in the Australian and Indonesian subspecies of the black-tailed rat (*Melomys burtoni*). This virus shares 93% sequence similarity with GaLV and 83% with KoRV [123]. The habitat of *Melomys burtoni* overlaps with that of koalas [123], and another study identified a subspecies of *Melomys burtoni* in Indonesia, the habitat of gibbons, with GaLV detected in the genome of this species [124,125]. These findings suggest that cross-species transmission between KoRV and GaLV may have occurred via MbRV in *Melomys burtoni*. Additionally, bats are believed to play a crucial role in this transmission process. Through metagenomic and phylogenetic analyses, researchers identified five ERVs carried by three bat species from Australia (*P. alecto*, *M. minimus*, and *S. australis*) and two bat species from Asia (*H. larvatus* and *R. hipposideros*), which cluster on the same branch or ancestral branches of the phylogenetic tree with KoRV and GaLV [126,127]. Further analysis revealed overlapping habitats of these bats between Asia and Australia [126], theoretically providing a pathway for the cross-species transmission of KoRV and GaLV.

In recent years, human activity-driven cross-species transmission of ERVs has become a significant topic in viral evolution. Recent surveys indicate that at least 73 vertebrate species carrying ERVs are closely linked to humans through various pathways. The primary transmission routes encompass multiple domains, including the food supply chain (e.g., livestock farming and consumption), pet ownership, clothing manufacturing, and artisanal collections [95]. Among these, 44 vertebrate species, through livestock farming or entry into the human food chain, may facilitate potential cross-species transmission of ERVs via digestive tract exposure, tissue ingestion, or blood contact during slaughter. Additionally, 43 species kept as pets or display animals may create sustained viral exposure windows through daily close contact with humans (e.g., hair, dander, or bodily fluid exchange). Notably, 39 vertebrate species (53.4%) are involved in two or more transmission pathways [95]. This multi-exposure pattern may significantly increase the likelihood of ERV contact with humans, thereby enhancing their cross-species transmission potential. Furthermore, the risk of ERV cross-species transmission during xenotransplantation has garnered significant attention. The pig genome contains three subtypes of PERV: PERV-A, PERV-B, and PERV-C [128]. PERV-A and PERV-B can directly infect human cells through the cell surface receptor HuPAR2 [129], while PERV-C requires recombination with PERV-A to acquire cross-species infection capability [130]. In vitro studies have demonstrated that ERV viral particles expressed by porcine cells can effectively infect human primary kidney cells, peripheral blood mononuclear cells, primary endothelial cells, and other cell types, exhibiting notable replication capacity [131]. However, no PERV infection events have been observed in pre-clinical xenotransplantation experiments or the first clinical trials to date [132]. These findings highlight the importance of investigating ERV transmission mechanisms, particularly in the context of frequent global human-animal interactions. A deeper understanding of their evolutionary effects on host species is critical for deciphering viral host adaptation and addressing potential public health risks.

## 8. Exaptation of ERVs in Host Immunity and Development

**Figure 2:**
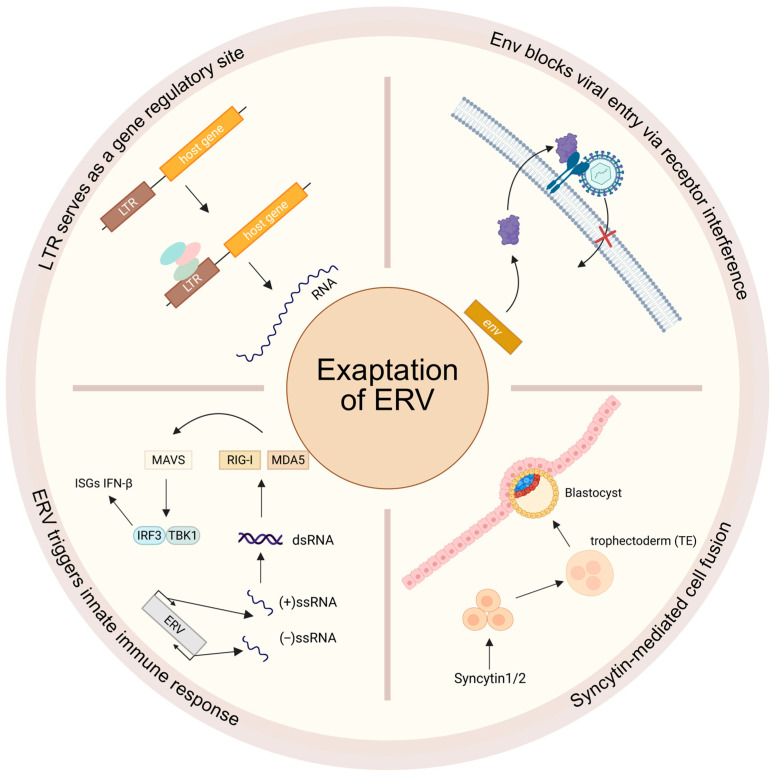
Exaptation of ERVs. LTRs can serve as transcription factor binding sites, promoters, or enhancers to regulate the expression of target genes; ERV-derived env genes can mediate immune interference by competitively occupying viral receptors; ERV-derived nucleic acid products are sensed by pattern recognition receptors such as RIG-I and MDA5, ultimately inducing the expression of IFN-β and a range of interferon-stimulated genes (ISGs); Syncytin-1/2 promote the fusion of trophoblast cells through their fusogenic activity, leading to the formation of multinucleated syncytiotrophoblasts.

Although ERVs are largely silenced by epigenetic mechanisms such as DNA methylation and histone modifications, their transcriptional activity can be reactivated under certain physiological or pathological conditions, including embryonic development, cellular senescence, and tumor transformation. The host utilizes these viral elements for various purposes, including regulating host gene expression, enhancing innate immunity and adaptive immunity [133], serving as key proteins for cell fusion during mammalian trophoblast development and promoting neural development (Figure 2) [133,134,135,136,137].

Activated ERVs can generate abundant virus-like nucleic acids, which play a critical role in the innate immune system [138]. These ERV-derived nucleic acid products are recognized by the innate immune system as “non-self” molecules, triggering a cascade of antiviral responses. Among them, cytoplasmic double-stranded RNA (dsRNA) is detected by pattern recognition receptors (PRRs) such as RIG-I and MDA5, leading to the polymerization and activation of the mitochondrial antiviral-signaling protein (MAVS). This pathway subsequently recruits TBK1 and IKKε, which phosphorylate interferon regulatory factors IRF3/7 and NF-κB, ultimately inducing the expression of type I interferons (e.g., IFN-β) and a range of interferon-stimulated genes (ISGs). These molecules establish an antiviral state within cells and activate neighboring cells via paracrine signaling, forming a broad antiviral barrier [139]. In addition to the classical RIG-I-like receptor pathway, certain ERV-derived reverse transcription products can also be recognized by cytoplasmic DNA-sensing systems, activating the cGAS-STING pathway, thereby further amplifying inflammatory responses and interferon production [140,141]. Notably, ERV transcription generates not only virus-like RNAs but also functional long non-coding RNAs (lncRNAs), which play important roles in immune regulation. For example, lnc-ALVE1-AS1 is an ERV-derived lncRNA that has been shown to directly bind Toll-like receptor TLR3 on endosomal membranes, thereby activating the classical NF-κB signaling cascade, inducing the secretion of proinflammatory cytokines such as IL-6 and TNF-α, and promoting the activation and chemotaxis of immune cells such as macrophages [142]. Other ERV-derived lncRNAs can bind to key transcription factors such as RELA, relieving their inhibitory states and further enhancing inflammatory and immune responses. The innate immune regulation mediated by lncRNAs not only expands the functional scope of ERVs in immune modulation but also reveals a complex and finely tuned regulatory network between non-coding RNAs and immune signaling pathways [143].

On the other hand, the long terminal repeats (LTRs) carried by ERVs not only function as promoters for ERV transcription but also exhibit significant cis-regulatory activity. LTRs are enriched in promoter and enhancer elements and contain multiple transcription factor binding sites, enabling recognition and activation by host transcription factors to regulate the expression of adjacent host genes. AIM2, an important cytosolic DNA sensor, is regulated by ERV/LTR (MER41B) and can initiate inflammasome assembly upon the recognition of exogenous DNA, leading to pyroptosis and IL-1β secretion, thereby enhancing immune clearance mechanisms [144]. In addition, nucleic acid fragments and protein components produced by ERV expression can be recognized by Toll-like receptors (TLRs) within the endosomal system, particularly TLR3 (recognizing dsRNA), TLR7 (recognizing ssRNA), and TLR9 (recognizing CpG-enriched DNA). Upon activation, these receptors signal through either the TRIF-dependent or MyD88-dependent pathways, leading to phosphorylation of IRF3/7 and activation of NF-κB, which induce the expression of numerous inflammatory cytokines, chemokines, and interferons. This establishes immune responses that span from the cellular level to systemic levels [145,146,147].

In the mouse genome, Fv-1 (Friend virus susceptibility 1) and Fv-4 (Friend virus susceptibility 4) are classic antiviral factors derived from ERVs, which exert specific inhibitory effects by targeting different critical stages of the retrovirus replication cycle. The Fv1 gene originates from a fragment of the *gag* gene of the mouse endogenous retrovirus (MuERV) [148], and its encoded product retains the capsid-binding domain of the ancestral Gag protein [148]. This protein specifically recognizes the MLV capsid protein and blocks the uncoating process of the viral core after the virus enters the host cell [149], thereby inhibiting the release of viral genomic RNA and subsequent reverse transcription activity [150,151]. The Fv-4 gene originated from the *env* gene of a defective ERV. Its encoded protein employs a molecular mimicry mechanism to competitively bind the murine leukemia virus (MLV) receptor (e.g., the cationic amino acid transporter CAT-1) on cell surfaces, thereby effectively blocking viral particle adsorption and membrane fusion. This process achieves pre-entry viral interception through immune interference [152]. Notably, a similar receptor interference mechanism has been observed in the formation and function of *Rmcf*. The recombinant Env proteins of *Rmcf*, derived from endogenous MLV sequences, target shared receptors such as Xpr1/SYG1. By competitively occupying these receptors, RMCF prevents subsequent infection by polytropic MLVs, establishing superinfection resistance [153,154]. This mechanism was first identified in DBA/2 mice and related inbred strains [153], and later extended to *Mus castaneus*, where a resistance gene termed *Rmcf2* was found to prevent P-MLV entry via a similar pathway [154]. These convergent strategies underscore the evolutionary significance of ERV-derived env genes in shaping antiviral defense and mediating interviral competition.

In mammals, various Env proteins with significant biological functions have been systematically analyzed. The most important and primary function of Env proteins is to promote cell fusion [155,156]. During early mammalian embryonic development, syncytins derived from *env*, such as Syncytin-1/2, facilitate the fusion of trophoblast cells through their cell fusion activity, forming multinucleated syncytiotrophoblasts [137,157]. This fusion is critical for the function of the embryonic trophoblast, as it helps form a structurally complex and functionally robust tissue to support placental development [158]. This mechanism represents a major evolutionary innovation in viviparous mammals, with independent exaptation of different syncytin analogs in primates, rodents, and other species [159]. Interestingly, in humans, the Syncytin-2 protein can also mediate immune tolerance at the maternal-fetal interface through its immunosuppressive domain, effectively inhibiting maternal immune rejection of the embryo [160]. Moreover, emerging studies have identified additional roles of syncytin proteins beyond placental development, particularly in cell–cell fusion processes in other tissues. One such example is the involvement of syncytin-1 and syncytin-B in osteoclast formation. These endogenous retroviral Env proteins are expressed during osteoclast differentiation and are implicated in the fusion of mononuclear precursors into multinucleated osteoclasts, a process essential for bone remodeling and resorption activity [161,162]. Syncytin-1, along with its receptor ASCT2, is upregulated in differentiating human osteoclasts and promotes the formation of large multinucleated cells (≥4 nuclei), while having limited effects on smaller osteoclasts (≤3 nuclei) [163]. Similarly, murine syncytin-B contributes to osteoclast precursor fusion; however, its genetic ablation does not significantly affect bone resorption, suggesting its role is primarily confined to facilitating fusion rather than functional activation [162]. These findings indicate that the fusogenic capabilities of syncytins have been repurposed during mammalian evolution to support diverse biological processes, including immune modulation and skeletal homeostasis, further underscoring their physiological significance beyond reproduction.

## 9. Conclusions and Prospects

ERVs are not only ancient remnants of viral infections but also important participants in the long-term co-evolution between hosts and viruses. Through mechanisms such as horizontal gene transfer, functional domestication, and immune regulation, they significantly influence the genomic plasticity and adaptive evolution of their hosts. The interactions between ERVs and XRVs, such as receptor competition and immune evasion, demonstrate the complex interplay between viruses and hosts. Cross-species transmission studies reveal the critical role of ERVs in species divergence and ecological adaptation, while regional distribution analyses provide molecular evidence for the migration history of host populations. Furthermore, functional proteins derived from ERVs, such as syncytins and antiviral factors, play important roles in embryonic development and immune defense. These findings not only deepen our understanding of viral-host coevolution but also provide new perspectives for disease mechanism research and biotechnological innovations.

Future research directions should address several critical gaps in our current knowledge. First, the strong bias toward mammalian systems necessitates expanded investigations in phylogenetically diverse vertebrates, particularly fish and amphibians, to uncover fundamental principles of retroviral-host coevolution. Second, the mechanisms governing ERV cross-species transmission remain poorly characterized beyond model systems like KoRV; comprehensive comparative genomics across taxonomic groups could reconstruct transmission networks and reveal the determinants of host switching. Third, the zoonotic potential of ERVs demands urgent attention through the development of predictive frameworks integrating machine learning and multi-omics data to assess spillover risks. Advanced genomic technologies, including single-cell and long-read sequencing, will enable the precise tracking of ERV dissemination through ecological networks. Most promisingly, the emerging field of paleovirology, through the temporal analysis of ERV integration patterns, promises to reconstruct ancient virus-host interactions, providing novel perspectives on both co-evolutionary processes and the deep history of viral diseases.

## Figures and Tables

**Table 1:** Types and classification of ERVs.

ERV Type	Retrovirus Type	Species	Virus
Class I ERVs	γ-retroviruses	*Ceratotherium simum simum*	SimumERV [14]
		*Felis silvestris catus*	ERV-DC8/FeLV [15]
	ε-retroviruses	*Eptatretus burgeri*	EbuERV [16]
Class II ERVs	α-retroviruses	*Gallus gallus*	ALVE [17]
	β-retroviruses	*Homo sapiens*	HML-2/HERVK [18]
		*Tasmanian devil*	MEBrv1/2/4 [19]
	δ-retroviruses	*rhinolophid bats*	ChirDelta2 [20]
		*Miniopterus natalensis*	MINERVa [21]
	lentiviruses	*Pedetes capensis*	SpELV [22]
		*Oryctolagus cuniculus*	RELIK [23]
Class III ERVs	spumaviruses	*Daubentonia madagascariensis*	PSFVaye [24]
		*Latimeria chalumnae*	CoeEFV [25]
		*Xenopus tropicalis*	XtERV-S [26]

**Table 2:** Species and pathways of ERV cross-species transmission.

Names of ERVs in Original Host Species	Original Host Species	Names of ERVs in New Host Species	New Host Species	Transmission Pathways	Nucleotide Similarity
RfRV	*Tupaia belangeri*	RfRV	*Pangolin*	Bats or rodents	Not reported [116]
enJSRV	*Ovis aries*	enJSRV	*Capra hircus*	Horizontal transmission	90% [118]
MLERV1	*Myotis lucifugus*	FcERV_γ6	*Felis catus*	Direct contact or predation	85% [119]
			*Panthera tigris*		
MLERV1	*Myotis lucifugus*	MPERV1	*Manis pentadactyla*	Direct contact	85% [119]
ERV-Spuma-Spu	*Sphenodon punctatus*	ERV-Spuma-Ppi ERV-Spuma-Gja	*Paroedura picta*	Unknown non- reptile host	Not reported [120]
			*Gekko japonicus*		
RnERV-K8e	*Rattus norvegicus*	MmERV-K10c	*Mus musculus*	Not reported	Partial similarity [121]
